# Is a Magnetic-Manual Targeting Device an Appealing Alternative for Distal Locking of Tibial Intramedullary Nails?

**DOI:** 10.5812/atr.10638

**Published:** 2013-06-01

**Authors:** Lukas L. Negrin, Vilmos Vécsei

**Affiliations:** 1 Department of Trauma Surgery, Medical University of Vienna, Vienna, Austria

**Keywords:** Orthopedic Procedure, Fracture Fixation, Intramedullary Nailing

## Abstract

**Background:**

In order to enable a radiation-free, accurate and simple positioning of distal locking screws, a combined magnetic and manual targeting system has been developed by Sanatmetal®. Where a low-frequency magnetic field is initially used to detect the position of the first drill hole and three more holes can be found with a mechanical template.

**Objectives:**

Our cadaver study was performed to evaluate the accuracy and efficiency of this device.

**Materials and Methods:**

In two runs, 30 probands (group 1: 10 students; group 2: 10 residents; group 3: 10 attendings), none of who being familiar with the device, tested the radiation-free system using 60 intact cadaver tibias. Each proband performed the surgical procedure twice in succession.

**Results:**

Referring to the first attempts, 9.6, 7.2 and 7.1 minutes were the time periods required to insert the four distal screws and the relevant values for the second attempts were 8.6, 6.3 and 6.2 minutes; in both cases revealing a significant difference between group 1 and 2 and group 1 and 3. Furthermore, the mean values within each group indicated a significant decrease of the test duration. Out of the 240 drillings, only one failure (group 1) occurred, representing an accuracy of 99.58 %. Of the probands, 90 % rated the targeting device better than the free-hand technique and 77 % at least attested a high user-friendliness.

**Conclusions:**

Due to our satisfactory test results, the brief training, the steep learning curve and the radiation-free technique the new device has to be considered an appealing alternative for distal locking.

## 1. Background

Intramedullary interlocked nailing is a well-established, generally accepted, but also technically demanding standard procedure for the treatment of diaphyseal and some metaphyseal tibial fractures requiring intraoperative fluoroscopic guidance for the reduction of the fracture, the placement of the nail and in most cases for the fixation of the distal screws (1-3). Although the problem of proximal locking is already solved, distal locking needs further improvement (4). At present, the free-hand technique (5, 6) is the most popular method (7). Generally, a skilled surgeon and an experienced radio-technician are required for achieving accurate distal locking in the least possible time with the least possible exposure to radiation (4, 8-10). Even though a lead apron provides adequate protection to surgeon’s trunk and gonads (11), her/his hands are frequently directly exposed to the x-ray beam. Per intramedullary nailing they receive a mean radiation dose of 0.330 mSv. Of interest, a value of 0.023 mSv is related to distal locking when performed by a consultant, whereas this value increases to 0.028 mSv for a middle-grade surgeon (9). Due to the fact that distal locking is a challenging step during intramedullary nailing the free-hand technique can lead to a significant increase in radiation exposure. As a result of deformation of the nail during its insertion into the intramedullary canal, a simple aiming arm, mounted on the proximal nail end alone is not adequate for the aiming process. Krettek et al. (12-14) analyzed implantation-induced nail deformation in unreamed tibial nails, detecting lateral translations in the range of 14.3 mm, dorsal translation in the range of 19.2 mm and rotational deformation around the longitudinal axis of the nail in the range of 2.4°. Without compensation for this deformation, it is impossible to place the screws accurately. Recently, an innovative radiation-independent aiming device for distal locking of intramedullary nails was presented by Sanatmetal®(Hungary, Eger).

## 2. Objectives

The objective of our study was to assess the Sanatmetal®distal targeting system’s effectiveness and accuracy in a human cadaver model, evaluating the duration of time taken for tibial distal locking, the accuracy of the screw targeting and the usability of the device in three different groups.

## 3. Materials and Methods

**Figure 1.**
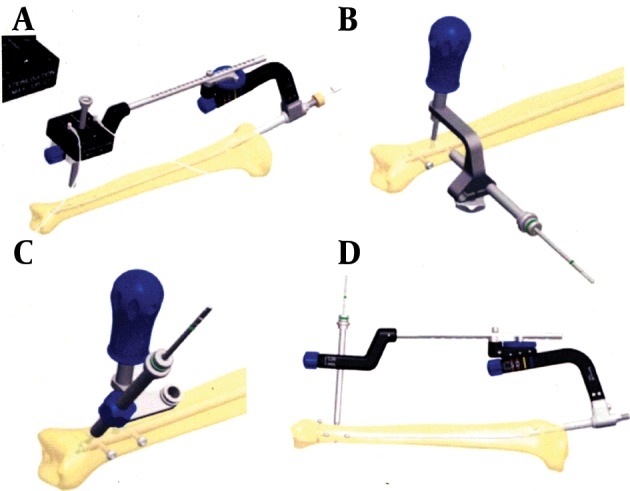
Sanatmetal Distal Targeting System

The magnetic targeting system is connected to an external power supply. It consists of a pivot-mounted distal targeting arm with the computer box and the proximal insertion guide connected to the nail (see Figure 1 a).

**Figure 2.**
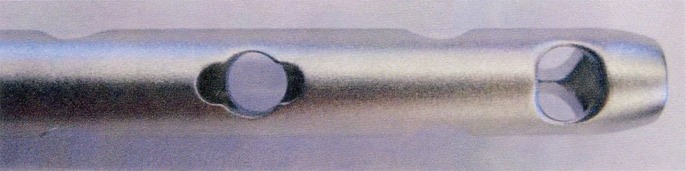
View of the Special Hole and the Two Holes in a V-Shaped Position at the Distal End of the Nail; the Two Perpendicular Holes for Lateral Interlocking Can Also Be Identified

The computer box (it can be sterilized at 134 ºC and –0.5 to +2.0 atm pressure) includes a signal source coil; It creates a low-frequency (2 kHz) magnetic field (source field) which induces an electric field in the receiver coil (sensor). The sensor is inserted into the intramedullary nail and then connected to the computer box. Furthermore, the source field induces eddy currents in the nail, provoking magnetic fields themselves which superpose the source field. Due to the symmetric design of the so-called special hole of the inserted SpectruM® tibia nail (see Figure 2), located near its distal end, destructive interference occurs if the signal source is directly placed above it.

This means that the source field and the overall magnetic field of the eddy currents cancel each other, resulting in an almost zero electric field in the receiver coil. A red light-emitting diode on the surface of the box indicates the voltage (= strength of the electric field) of the receiver coil; it turns off at (almost) zero, displaying the correct position of the distal targeting arm. After performing the skin incision and placing the soft-tissue protection sleeve onto the bone, the correct position can be verified again. Then the sensor has to be pulled out and the drill can be driven into the first corticalis that lies directly over the special hole of the nail. Following this process, the distal targeting arm is removed and the manual device with a 90 degree arm is applied. An appropriate touch probe is put into the special hole until it is securely locked into place. Two notches for the drill sleeve enable the drill to penetrate the respective lateral locking holes (Figure 1 b). For fractures that are located very distally with a short bone stock, the manual targeting device provides two holes in V shaped positions, which are placed in the very distal end of the nail (Figure 2). With the touch probe remaining in the special hole, the 90 degree arm is replaced by a 30 degree arm enabling the setting of an oblique screw either from medial or lateral, dependent on its montage (Figure 1 c). For special indications the distal targeting arm without the computer box can be fixed on the insertion guide again, in order to place an additional screw in a sagittal plane (Figure 1 d). The drill sleeve can be directly inserted into the special hole if the setting of the dial was not changed. In case it was changed, the hole can easily be found again in order to penetrate the second corticalis. Finally, proximal locking can be performed similar to commonly used systems. Due to body donations, 60 intact cadaver tibias without a fracture were available for the authors. They were used to test the Sanatmetal® distal targeting device for implanting the SpectruM® tibia nail. We evaluated its accuracy and the time period that was necessary to place all of the four provided distal screws, starting with the montage of the distal targeting device onto the insertion guide. 30 probands, none of them familiar with the device at the time of investigation were subdivided into three equal groups. Group I consisted of students who are interested in trauma surgery and understand the principles of osteosynthesis, but are without any practical experience. 3rd-year-residents or higher who have performed the free-hand technique for several times formed group 2, whereas board certified trauma surgeons/attendings were combined to form group 3. Each of them performed the surgical procedure twice in succession, after having received an introductory training by the first author; he also played the role of the surgical nurse during the experiment. After completing the test series, each participant had to answer two questions. The relevant opinion had to be quantified on a scaled form, with 1 being very good and 5 being very bad. Question 1: To your opinion, how important is the availability of a distal targeting system without radiation exposure? Question 2: How user-friendly is the utilized Sanatmetal® device? Finally, an additional question was raised to the residents and the attendings that had to be rated on a scale of five values (1: much better, 3: equal, 5: much worse). Question 3: How does the Sanatmetal® device perform compared to the free-hand technique? All statistical analyses were done using IBM SPSS statistics 20.

## 4. Results

**Figure 3.**
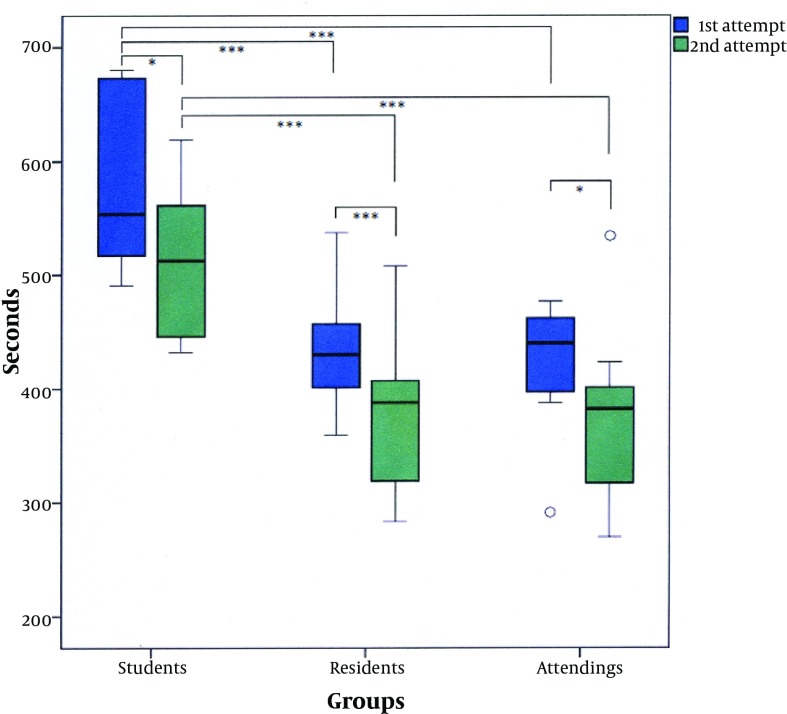
Graphical Overview of the Test Durations *, P value of < 0.05; ***, P value of < 0.0001

The students required 574 ± 75 seconds to insert the four distal screws during their first attempt and 515 ± 68 seconds for their second attempt. The residents needed 434 ± 51 seconds and 376 ± 65 seconds, whereas 426 ± 56 seconds and 373 ± 77 seconds were sufficient for the attendings. With P = 0.025, < 0.0001 and 0.012 respectively, the mean values within each group revealed a significant decrease in the test duration. For the first attempt, the residents were significantly faster than the students (P < 0.0001), whereas there was no significant difference when compared to the attendings (P = 0.719). The attendings, not surprisingly, performed the intramedullary locking significantly faster than the students (P < 0.0001). Comparing the second attempt of the probands, there was a significant difference between the students and the residents as well as between the students and the attendings (P < 0.0001). However, no significant difference was revealed between the residents and the attendings (P = 0.918). The medians and the range of the relevant time periods needed for inserting the four distal screws are graphically displayed by boxplots, presented in Figure 3.

In the second run almost all probands were faster, except for two students and one attending who needed more time (5 seconds, 40 seconds and 1 minute respectively). Out of 240 drillings, only one miss drilling (group 1) occurred due to the fact, that the touch probe was not securely locked into the special hole. Therefore, the test results represent an accuracy of 99.58 %. After performing the tests, each proband was asked two questions. For question 1, dealing with the necessity of a distal targeting device without radiation exposure, was predominately answered in the affirmative, as revealed by Table 1. The user-friendliness of the Sanatmetal® distal targeting system (question 2) was considered at least “moderate” (Table 2). Furthermore, 18 out of 20 residents and attendings (90 %) rated the Sanatmetal® targeting system better than the free-hand technique (Table 3).

**Table 1.** Distribution of Answers Assessing the Need of a Distal Targeting System Without Radiation

	Very Important	Important	Equal	Rather Unimportant	Unimportant
**Students**	7	3	0	0	0
**Residents**	6	3	1	0	0
**Attendings**	6	2	1	1	0
**Total**	19	8	2	1	0

**Table 2.** Distribution of Answers Assessing the User-Friendliness of the Sanatmetal^®^ Distal Targeting System

	Very High	High	Moderate	Rather Low	Very Low
**Students**	5	5	0	0	0
**Residents**	2	5	3	0	0
**Attendings**	1	5	4	0	0
**Total**	8	15	7	0	0

**Table 3.** Distribution of Answers, Comparing the Sanatmetal^®^ Distal Targeting System to the Free-Hand Technique

	Much Better	Better	Equal	Worse	Far Worse
**Residents**	3	6	1	0	0
**Attending**	2	7	1	0	0
**Total**	5	13	2	0	0

## 5. Discussion

Intramedullary nailing is considered one of the most technically demanding procedures in orthopedic trauma surgery (4, 8, 10). Using the free-hand technique, a varying number of x-ray images is necessary to locate the axes of the distal holes on the intramedullary nail depending on the surgeon’s skills. Generally, the International Commission on Radiological Protection (ICRP) recommends a maximum permissible dose of 500 mSv per year in the case that only a single portion of the body (any individual organ or tissue other than the lens of the eye) is exposed to radiation (15). Nevertheless, radiation exposure should be kept as low as reasonably achievable because the long-term effects of low-level radiation and their relationship to different types of cancer are still largely unknown (16, 17). According to the linear no-threshold theory (18) ionizing radiation, no matter how small, may provoke adverse effects after many years. Therefore, to our opinion, aiming devices for distal locking of intramedullary nails without the need of x-ray control have to be welcomed on principle. Nevertheless, a new technique will only prevail against the free-hand technique and other targeting systems, if it is precise, reliable and easy to apply. Misplacement of distal interlocking screws can lead to iatrogenic fractures, instability of the bone-implant construct or even misalignment of the extremity; repeated drilling attempts can cause additional bony and soft tissue trauma (19). The Sanatmetal® targeting system is quite simple and easy to learn; our cadaver study revealed a mean duration of 9.6 minutes at the most for the setting of four distal screws. Of interest, the mean targeting time (time between the beginning of alignment of the distal holes and setting of the two distal screws) of a mechanical system needing fluoroscopy that was applied in a clinical trial was evaluated to be 24 (20 to 30) minutes (20); nevertheless, taking into account all of the differences between our cadaver and this clinical study, we could show that the Sanatmetal® device is able to assist surgeons in rapid drilling of holes for distal locking screws. The system is sufficiently robust and profoundly precise; only one failure occurred due to a surgical error of a student. Of the probands, 90 % rated the Sanatmetal®targeting device better than the free-hand technique and 77 % at least attested a high user-friendliness. Limitations of our study include; a) the fact that our tests could not be performed on the entire lower extremities of fresh human cadavers, b) no fracture model was applied and c) the number of available cadaver tibias did not enable a third run for the probands. Nevertheless, our cadaver study indicates that the Sanatmetal® distal targeting system is reliable, accurate and user-friendly; therefore, it is helpful especially for inexperienced hands. Due to our satisfactory test results, the brief training, the steep learning curve and the radiation-free technique, the Sanatmetal®targeting device has to be considered as an appealing alternative for distal locking.
